# Transparent Polyaniline Thin Film Synthesized Using a Low-Voltage-Driven Atmospheric Pressure Plasma Reactor

**DOI:** 10.3390/ma14051278

**Published:** 2021-03-08

**Authors:** Jae Young Kim, Shahzad Iqbal, Hyo Jun Jang, Eun Young Jung, Gyu Tae Bae, Choon Sang Park, Bhum Jae Shin, Heung Sik Tae

**Affiliations:** 1 School of Electronic and Electrical Engineering, College of IT Engineering, Kyungpook National University, Daegu 41566, Korea; jyk@knu.ac.kr (J.Y.K.); shahzadiqbal@knu.ac.kr (S.I.); bs00201@knu.ac.kr (H.J.J.); eyjung@knu.ac.kr (E.Y.J.); doctor047@knu.ac.kr (G.T.B.); 2 Department of Electrical and Computer Engineering, College of Engineering, Kansas State University, Manhattan, KS 66506, USA; purplepcs@ksu.edu; 3 Department of Electronics Engineering, Sejong University, Seoul 05006, Korea; hahusbi@sejong.ac.kr; 4 School of Electronics Engineering, College of IT Engineering, Kyungpook National University, Daegu 41566, Korea

**Keywords:** aniline, atmospheric pressure plasma reactor, conjugated polymer film, plasma polymerization, polyaniline

## Abstract

The use of low-voltage-driven plasma in atmospheric pressure (AP) plasma polymerization is considered as a simple approach to reducing the reactivity of the monomer fragments in order to prevent excessive cross-linking, which would have a negative effect on the structural properties of the polymerized thin films. In this study, AP-plasma polymerization can be processed at low voltage by an AP-plasma reactor with a wire electrode configuration. A bare tungsten wire is used as a powered electrode to initiate discharge in the plasma area (defined as the area between the wide glass tube and the substrate stand), thus allowing plasma polymerization to proceed at a lower voltage compared to other AP-plasma reactors with dielectric barriers. Thus, transparent polyaniline (PANI) films are successfully synthesized. The surface morphology, roughness, and film thickness of the PANI films are characterized by field emission scanning electron microscopy and atomic force microscopy. Thus, the surface of the polymerized film is shown to be homogenous, smooth, and flat, with a low surface roughness of 1 nm. In addition, the structure and chemical properties of the PANI films are investigated by Fourier transform infrared spectroscopy, thus revealing an improvement in the degree of polymerization, even though the process was performed at low voltage.

## 1. Introduction

Plasma polymerization refers to the synthesis of thin films with very high cross-linking density on the surface of a substrate via the gaseous discharge of organic monomer vapors [1,2,3,4,5]. Plasma polymerized films have been deposited from chemical monomers containing atoms that create polymer chains, such as carbon, sulfur, and silicon [6,7,8,9,10]. Most of the monomer molecules introduced into the plasma medium are broken into reactive species by the plasma energy, while a partial chemical structure of the monomer is preserved and cross-linked, thus resulting in irregular structures of the polymerized films. These structures and physicochemical properties can be precisely controlled by adjusting various experimental parameters of the generated plasma, such as the configuration of the plasma device, the type of discharge gas, the gas flowrate, the plasma driving conditions (voltage and current waveforms), and any additional voltage bias.

The present authors have recently published several reports on the plasma polymerization method using atmospheric pressure (AP) plasma reactors [11,12,13,14,15,16,17]. For the stable generation of glow plasma at AP, a plasma jet device with a dielectric barrier was introduced, and three quartz tube jet devices were used in the form of an array to uniformly deposit a polymer thin film on a substrate of several square centimeters in area. A guide tube and a bluff body were additionally installed into this plasma array device to maintain the spatial uniformity of the discharge gas containing the monomer vapor in the polymerization reaction area for a longer time. This AP-plasma jet (APPJ) array was easily able to maintain a stable glow discharge because the discharge current was automatically controlled by the dielectric barrier [11,12,13,14,15]. However, high AC voltages were required to charge and discharge the capacitive structures via the dielectric barrier. Consequently, the relatively high plasma energy generated at the high applied voltage led to excessive cross-linking of the highly reactive monomer species during AP polymerization, thus resulting in polymerized films with irregular structures, rough morphological features, and a lack of optical transparency, thereby limiting the scope of this approach for various applications.

To control the structural properties of the polymer thin films synthesized via AP-plasma polymerization, it is necessary to change the plasma polymerization conditions drastically. In particular, it is expected that lowering the driving voltage can provide a novel approach to obtaining polymerized films with new functional properties. If the polymerization is performed using a relatively low plasma energy (low driving voltage), excessive and random cross-linking of the monomer species can be avoided, and the irregular cross-linked structure of the polymerized film might be replaced by a regular and smooth structure. More recently, the present authors have developed a new type of AP-plasma reactor for advanced in-situ iodine doping process that can improve the electrical properties of conjugated polymers [18]. This AP-plasma reactor generated a stable gaseous discharge at a low applied voltage using a bare wire electrode exposed to the plasma area.

Accordingly, in this study, a low-voltage-driven plasma polymerization process is performed using this AP-plasma reactor for the purpose of controlling the structural properties of polymer thin films. The discharge characteristics of the resulting plasma are investigated by monitoring the voltage, current, and optical emission waveforms on an oscilloscope. Using this newly designed AP-plasma reactor, the amount of monomer vapor for polymerization can be increased while obtaining a stable glow plasma state. As a result, polyaniline (PANI) films with good transparency are obtained at a low applied voltage of 4 kV. In addition, the PANI thin film deposited from a solution of the aniline monomer is characterized in detail via ultraviolet-visible near-infrared (UV-vis-NIR) spectrophotometry, field emission scanning electron microscopy (FE-SEM), atomic force microscopy (AFM), and Fourier transform infrared spectroscopy (FT-IR).

## 2. Materials and Methods

### 2.1. The AP-Plasma Polymerization System

**Figure 1:**
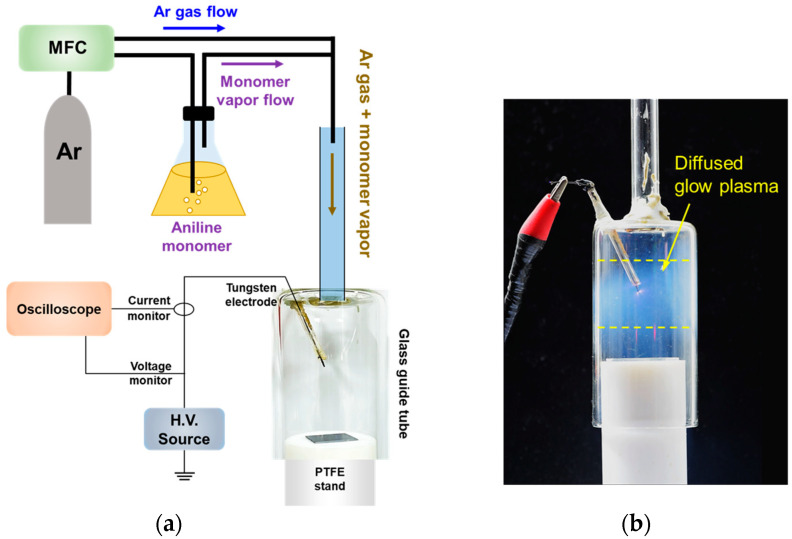
The atmospheric pressure (AP) plasma polymerization setup employed in this study: (**a**) schematic diagram of the AP-plasma polymerization system and (**b**) photograph of the AP-plasma reactor during plasma polymerization.

The newly proposed AP-plasma reactor and the entire AP-plasma polymerization system employed in this study were described in detail in our recent previous work [18] and schematically shown in Figure 1a. Compared to the previous work, the entire system was simplified because an iodine container is not required for in-situ doping. Argon (Ar) gas with a purity of 99.999% was used as the discharge gas at a flow rate of 1300 standard cubic centimeters per minute (sccm). Aniline monomers with an average molecular weight of MW = 93 g/mol vaporized by Ar gas was supplied into the AP-plasma reactor at a flow rate of 400 sccm. Using the inverter circuit, a sinusoidal voltage with a peak value of 4 kV and a frequency of 30 kHz was applied to the AP-plasma reactor.

### 2.2. Characterizations of the AP-Plasma and the Deposited Polyaniline Thin Film

To observe the electrical characteristics of the produced generated plasma, the voltage and current waveforms from the powered electrode were monitored using a high-voltage (HV) probe (P6015A, Tektronix Inc., Beaverton, OR, USA) and a current transformer (4100, Pearson Electronics Inc., Palo Alto, CA, USA).

A photo-sensor amplifier (C6386-01, Hamamatsu Corp., Hamamatsu, Japan) and optical fiber-based compact spectrometer (USB-2000+, Ocean Optics Inc., Dunedin, FL, USA) were used to observe the plasma emissions. The optical characterization methods of the generated AP-plasma were described in detail in a previous report [18].

The surface morphology and thickness of the PANI films on glass substrates were characterized by cross-sectional FE-SEM imaging (SU8220, Hitachi High-Technologies, Tokyo, Japan) with accelerated electrons at a voltage of 3 kV and a current of 10 mA. Prior to taking FE-SEM images, the PANI film samples were coated with platinum to avoid surface charge problems.

The surface roughness of the PANI thin films was examined using three-dimensional topographical images obtained by non-contact mode AFM (Brucker, NanoWizard II, Ettlingen, Germany) at the Korea Basic Science Institute (KBSI, Busan, Korea) with a 50 × 50 μm (256 × 256 pixel) scanning area and a scan rate of 1 Hz. All the measurements were obtained under controlled room temperature and the data were processed and interpreted using the Bruker NanoWizard software.

The chemical structures of the various PANI thin films were identified via FT-IR (Vertex 70, Bruker, Ettlingen, Germany) at the Korea Basic Science Institute (KBSI, Daegu, Korea). The FT-IR spectra were obtained by the average of 128 scans at a wavenumber resolution of 0.6 cm^−1^ in the range of 650–4000 cm^−1^ using the attenuated total reflection mode.

The optical transmittances of the PANI thin films on the glass substrates were measured using a UV-vis-NIR spectrophotometer (Lambda 950, PerkinElmer, Waltham, MA, USA) at the Korea Basic Science Institute (KBSI, Daegu, Korea).

## 3. Results and Discussion

### 3.1. The AP-Plasma Reactor with a Bare Wire Electrode

In general, APPJs have a simple structure consisting of a gas-feeding tube and electrodes. To prevent the unwanted transition of glow to arc discharge, a dielectric barrier that can easily control the current during discharge is commonly used, thus resulting in AC discharge [19,20,21,22,23]. When generating a DC discharge, it is common to expose the powered electrode to the discharge space, include a proper ballast resistor in the circuit to control the current, and ground the counter electrode [24,25,26,27,28,29,30,31]. However, even when the tip of the tungsten wire electrode of the newly designed AP-plasma reactor was exposed to the discharge space, a ballast resistor was not needed because the AC voltage was applied at a frequency of about 30 kHz, and the glow discharge occurred in a single electrode structure without a ground electrode.

The combination of a guide tube and bluff body was proposed for AP-plasma polymerization in previous reports by the present authors [11,12,13,14]. The guide tube and bluff body were installed at the end of the APPJ array and were not intended for plasma generation but for maintaining a widely spaced plasma over an extended time in order to form a uniform polymer thin film. However, the direct application of a voltage to the exposed wire electrode transformed the space between the wide tube and PTFE stand into both a plasma generation and polymerization reaction space as shown in Figure 1b [18]. In particular, the spatial separation of the voltage application region from the gas-emanating region enables the plasma polymerization to proceed near the substrate, thus resulting in the efficient fabrication of the polymer thin film.

### 3.2. Electrical and Optical Properties of the Plasma Produced by the Newly Designed Plasma Reactor

**Figure 2:**
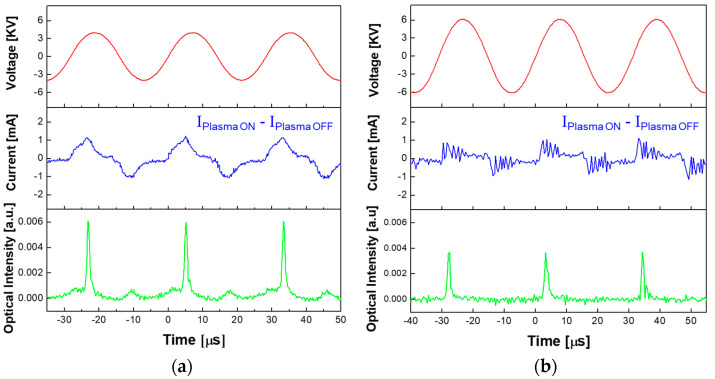
The temporal characteristics of driving voltage, discharge current, and optical emission from the plasmas produced using (**a**) the newly designed AP-plasma reactor, and (**b**) the previously reported APPJ array. The discharge current waveform was obtained by subtracting the displacement current waveform from the total current waveform.

To identify the discharge behavior of the newly designed AP-plasma reactor, the discharge current and optical emissions were each measured as a function of time, and the results are presented in Figure 2a. For comparison, the corresponding characteristics of the previously reported APPJ array are presented in Figure 2b. In each case, the discharge current waveform was obtained from the difference between the current waveforms obtained when the plasma was turned off and when it was turned on. Details of the experimental conditions for plasma polymerization using the previously reported APPJ array and the newly designed AP-plasma reactor are summarized in Table 1.

The discharge current waveform obtained using the newly designed AP-plasma reactor (middle panel, Figure 2a) indicates that each discharge event occurs during the rising and falling periods of the voltage waveform (top panel, Figure 2a), thus demonstrating successful discharge even with a single electrode serving as both anode and cathode. In addition, the temporal behavior of the optical emissions measured near the wire electrode in the plasma polymerization area (bottom panel, Figure 2a) is seen to be periodically stable, and the optical intensity during the rising slope of the voltage waveform is higher than that during the falling slope. The optical intensity results in Figure 2a,b indicate that stronger plasmas are generated when the single electrode serves as the anode.

Since the newly designed AP-plasma reactor can generate plasma at a lower voltage, the optimal driving voltage for polymerization is correspondingly much lower than that of the previously reported AP-plasma devices [11,12,13]. In the newly designed AP-plasma reactor, the glow discharge initiates at an applied voltage of 2.8 kV_P_ (peak voltage), and the driving voltage for stable AP-plasma polymerization is as low as 4 kV_P_, which is half that of the previously reported APPJ array. Thus, the newly designed AP-plasma reactor allows the amount of monomer vapor for polymerization to be increased while obtaining a stable plasma state. In addition, because the newly proposed AP-plasma reactor does not have a dielectric barrier which is a capacitive component, the displacement current part is relatively small compared to the previously reported APPJ array. Therefore, the average power dissipated in the newly proposed AP-plasma reactor is 12 W, which is approximately 25% of the average power of the previously reported APPJ array as shown in Table 1. The shapes of the discharge current waveforms in Figure 2a,b are also seen to be quite different. In the conventional AP-plasma array device, the discharge current waveform exhibits a series of narrow peaks due to control of the current by the dielectric barrier between the electrode and the discharge space. By contrast, the discharge current waveform in the newly designed AP-plasma reactor is maintained for a certain period of time, and the discharge occurs continuously for that time.

**Figure 3:**
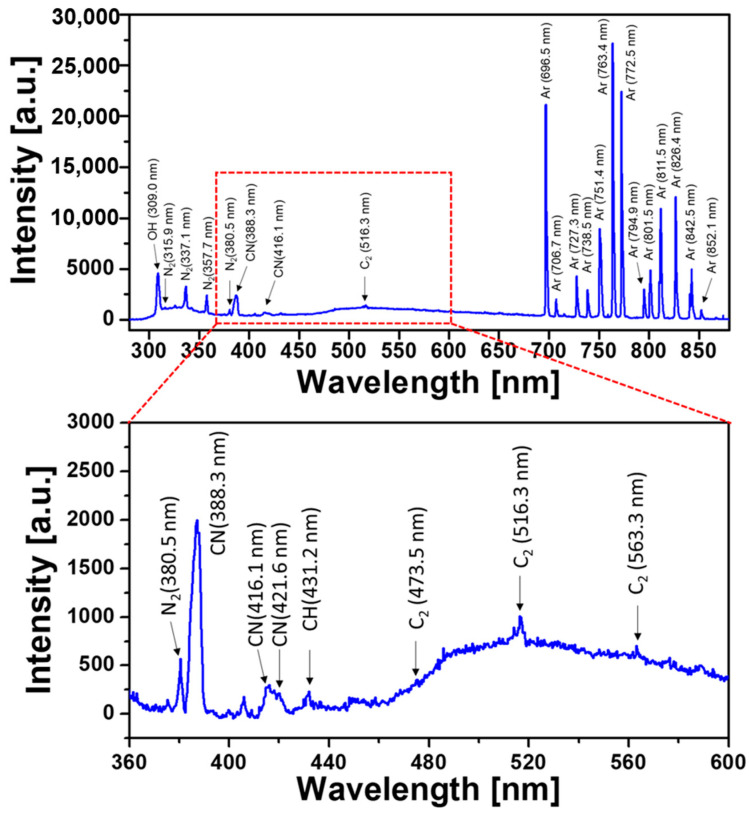
Optical emission spectra (OES) measured during polymerization reaction.

The reactive species produced by the newly designed AP-plasma reactor are revealed by the optical emission spectra acquired during polymerization procedure. Figure 3 shows the emission spectra ranging from 300 to 875 nm, indicating that the excited OH, N_2_, Ar, and carbon-related species exist in the plasma-generating region. For better visualization to identify transition lines in the carbon fragments from aniline monomer vapor, the range of 360 to 600 nm is magnified as shown in the lower graph of Figure 3. Transition lines from carbon fragments are present in the optical emission spectrum of the AP-plasma polymerization system: CN bands at 388.3, 416.13, and 421.6 nm, a CH band at 431.2 nm, and C_2_ lines at 473.5, 516.3, and 563.3 nm. The presence of these transition lines associated with carbon fragments confirms that aniline vapors are partially fragmented by plasma energy during plasma polymerization.

### 3.3. Characterization of the AP-Plasma Polymerized Aniline Thin Films

**Figure 4:**
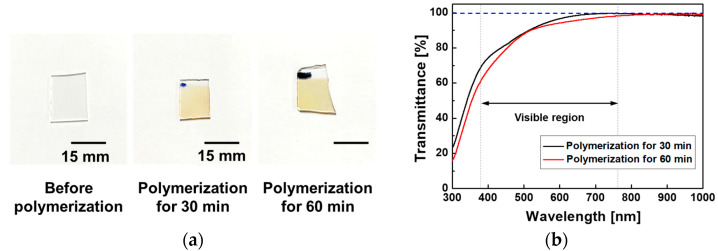
Characterization of transparent polyaniline (PANI) thin films obtained by polymerization on glass substrates for 30 min and 60 min: (**a**) photographic images, and (**b**) the UV-vis optical transmittance spectra.

The newly designed AP-plasma polymerization system was used to deposit the PANI-film glass substrates for 30 min and 60 min, as shown in Figure 4a. The diameter of the substrate stand of the AP-plasma reactor increased from 15 mm to 30 mm as depicted in Table 1. Thus, the maximum deposition area of the polymer thin film could be increased four times from 1 cm × 1 cm to 2 cm × 2 cm. As can be seen in Figure 4a, the area of the PANI thin film sample is approximately 15 mm × 15 mm due to the experimental margin, but it is actually increased in comparison with the previous reports [11,13]. As the growth rate of the PANI thin film on amorphous substrates such as glass is relatively slow, relatively transparent films are obtained. The UV-vis-NIR optical transmittance spectra in Figure 4b indicate a high optical transmittance of 90% in the visible wavelength region of 500 to 700 nm, thus explaining the transparent nature if the PANI films, which appear slightly amber when observed with the naked eye (Figure 4a).

**Figure 5:**
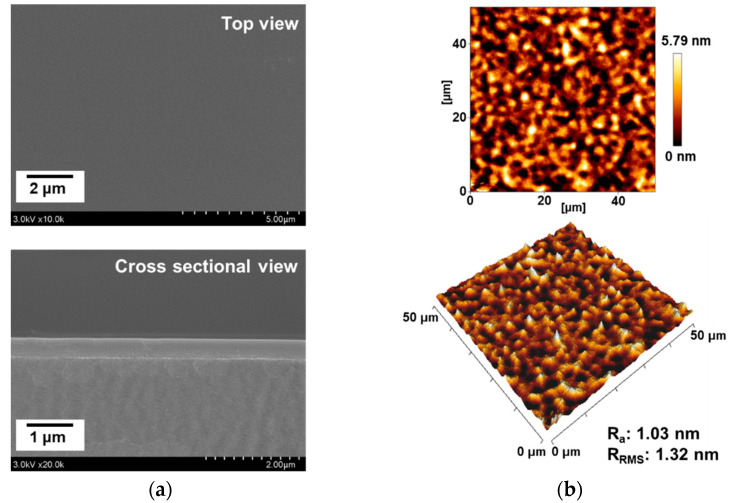
Surface characterization of the PANI film obtained on a glass substrate by using the newly designed AP-plasma reactor system for 60 min: (**a**) top-view and cross-sectional view of FE-SEM images; (**b**) two-dimensional and three-dimensional AFM images.

Furthermore, the top view and cross-sectional FE-SEM images of the PANI thin film obtained by 60 min polymerization on the glass substrate (Figure 5a) clearly reveal a homogenous, smooth, and flat surface with a thickness of ~450 nm, which is quite distinct from the previously reported results using the APPJ with a high applied voltage [11,13,14]. In addition, the two- and three-dimensional AFM images in Figure 5b indicate an average surface roughness (R_a_) of 1.03 nm and a root mean square (R_RMS_) roughness of 1.32 nm.

Taken together, the UV-vis-NIR, FE-SEM, and AFM results indicate that when the AP-plasma polymerization is processed at a lower voltage, the reactive monomers do not have energy enough to undergo excessive random cross-linking. As a result, the reactive monomer species are uniformly stacked and undergo stable cross-linking in order of arrival at the substrate from the plasma region, thus forming a smooth and flat thin film (Figure 5). Therefore, the resulting PANI layer is sufficiently structurally dense to be transparent, as shown in Figure 4.

**Figure 6:**
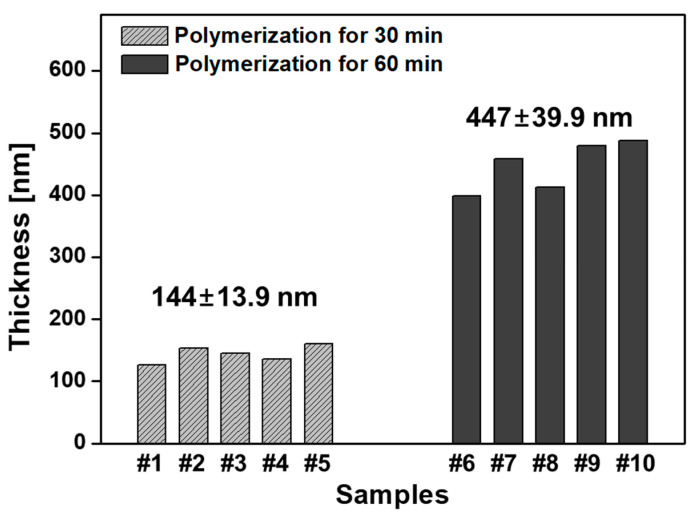
Thicknesses of PANI films grown on glass substrates by using the newly designed AP-plasma reactor system. Samples #1 to # 5 were polymerized for 30 min, and samples #6 to #10 were polymerized for 60 min.

In order to investigate the repeatability and reproducibility of the resultant PANI thin films, the proposed AP-plasma polymerization system was used to fabricate total ten PANI films on glass substrates for 30 and 60 min (five samples for each). As shown in Figure 6, the PANI thin films with similar thicknesses are deposited during each polymerization time, 30 and 60 min, and the corresponding thicknesses are measured to be approximately 145 and 450 nm, respectively. The thicknesses shown in Figure 6 are the means ± standard deviations (SDs) from ten PANI films after polymerizations for 30 and 60 min. Therefore, it is confirmed that transparent PANI thin films can be stably and repeatedly fabricated with this AP-plasma polymerization method.

**Figure 7:**
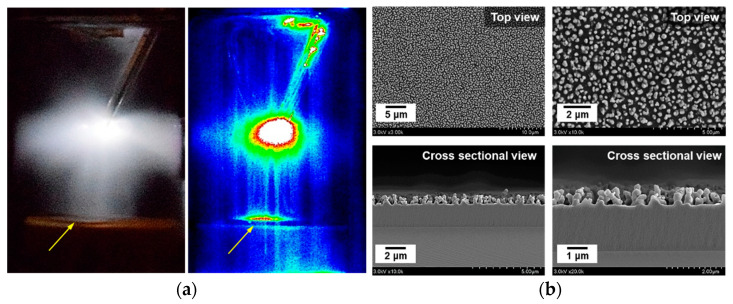
AP-plasma polymerization in the plasma coupling mode: (**a**) digital photograph and ICCD image of the generated glow discharges, and (**b**) SEM images of the resulting PANI film.

When AP-plasma polymerization proceeds as the plasma generated from the electrode comes into direct contact with the substrate, this is termed the plasma coupling mode [15,16]. A digital photograph and an intensified charge coupled device (ICCD) image of the glow discharge generated in the plasma coupling mode are presented in Figure 7a. Here, the yellow arrows indicate where the generated glow plasma is in contact with the substrate. Furthermore, the SEM images in Figure 7b clearly indicate the highly homogeneous, crystal-like pattern on the surface of the PANI film that was formed in the plasma coupling mode. However, this pattern resulted in diffuse reflection and adversely affected the light transmission properties of the film. Hence, to avoid this issue, plasma coupling must be avoided by adjusting and optimizing the operating conditions (e.g., the plasma-driving conditions and the distance between the wire electrode and the PTFE stand) of the plasma polymerization. Moreover, since the newly designed AP-plasma reactor has no dielectric barrier, the more straight-forward approach is to control the distance between the electrode and the PTFE stand rather than precisely changing the driving conditions. In practice, the optimal distance between the electrode and the PTFE stand for avoiding plasma coupling and obtaining a transparent PANI film was found to be 30 mm.

**Figure 8:**
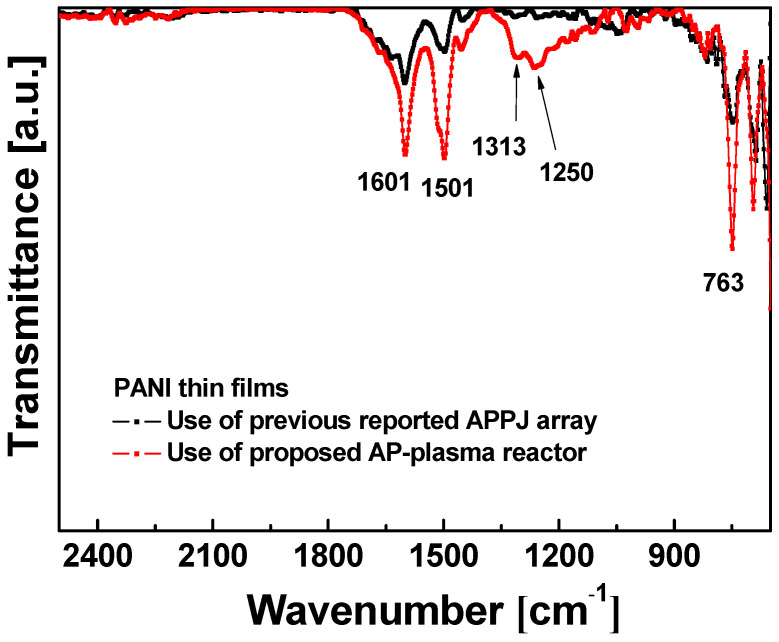
Comparison of the FT-IR spectra of the PANI thin films fabricated using the newly designed AP-plasma reactor (red line) and the previously reported APPJ array (black line).

The FT-IR spectra of PANI thin films prepared using the newly designed AP-plasma reactor and the previously reported APPJ array are presented in Figure 8. Here, the FT-IR spectrum of the film deposited using the newly designed reactor (red line, Figure 8) clearly exhibits the characteristic peaks of the PANI polymer structure at 1601, 1501, 1313, 1250, and 763 cm^−1^. In detail, the peaks at 1501 and 1601 cm^−1^ are attributed to the benzenoid and quinoid ring stretching vibrations, respectively; the peak at 763 cm^−1^ is ascribed to the C–H out-of-plane deformation in the aromatic ring, and the bands at 1250 and 1313 cm^−1^ are ascribed to the C–N stretching vibration [32,33,34,35].

The results in Figure 8 also indicate that the peak intensities of the conjugated bonds at 763, 1250, 1313, 1501, and 1601 cm^−1^ are increased for the film that was synthesized using the newly designed AP-plasma reactor (red line, Figure 8) compared to that deposited the previously reported plasma jet array (black line, Figure 8). This indicates an improvement in the degree of polymerization when using the new AP-plasma polymerization process, which is probably due to the use of Ar gas containing a greater amount of aniline monomer vapor compared to the previously reported plasma polymerization conditions (Table 1). Furthermore, the intensities of the C–N peaks at 1250 cm^−1^ and 1313 cm^−1^ are significantly increased for the film that was deposited using the new reactor compared to the previously reported plasma polymerization method. This C–N bond is strongly related to the electrical conductivity provided by the acidic proton released during N-conjugation of the quinone ring [36,37]. This increase in the number of conjugated bonds is expected to enhance the inter-molecular π–π stacking of the polymer chains, thus resulting in enhanced carrier mobility and good electrical conductivity [36,37].

## 4. Conclusions

In this study, a newly designed AP-plasma reactor capable of performing plasma polymerization at low voltage was described, and its application was investigated in the fabrication of polyaniline thin films. In the new reactor, the plasma is generated at a low voltage by a bare wire electrode exposed to the discharge area, thus enabling the generation of a stable glow plasma and successful plasma polymerization, even though the aniline monomer is vaporized with a higher Ar gas flowrate (400 sccm) than that used in previously reported procedures. This resulted in the synthesis of a homogeneous, transparent, and flat PANI thin film. A detailed investigation of the resulting PANI thin films provided key clues towards overcoming the performance limitations of the conventional APPJ array with the dielectric barrier. Thus, the new reactor design results in the deposition of conductive polymer thin films with enhanced transparency and, hence, a broadened range of application fields.

## Figures and Tables

**Table 1:** Summarized experimental conditions using the previously reported APPJ array reactor [11,13] and the newly designed AP-plasma reactor for AP-plasma polymerization.

Experimental Conditions		Previously Reported AP-Plasma Jet Array	Newly Designed AP-Plasma Reactor
Device Configuration	Electrode type	Single electrode	Single electrode
	Electrode material	Copper tape	Tungsten wire
	Use of dielectric barrier	Yes	No
	Diameter of wide tube	20 mm	34 mm
	Diameter of substrate stand	15 mm	30 mm
Driving Conditions	Driving type	AC	AC
	Voltage waveform	Sinusoidal	Sinusoidal
	Plasma initiation voltage (V_p_)	12 kV	2.8 kV
	Plasma driving voltage (V_p_)	8 kV	4 kV
	Driving frequency	26 kHz	30 kHz
	Average power dissipated (P_RMS_) ^1^	50.03 W	12.00 W
Gas Conditions	Gas type	Ar	Ar
	Gas purity	HP grade (99.999%)	HP grade (99.999%)
	Flow rate for discharge	1700 sccm	1300 sccm
	Flow rate for vaporization	160 sccm	400 sccm

^1^ Average power dissipated in plasma reactor is calculated by P_RMS_ = V_RMS_ × I_RMS_.
